# An analysis of simple computational strategies to facilitate the design of functional molecular information processors

**DOI:** 10.1186/s12859-016-1297-x

**Published:** 2016-10-28

**Authors:** Yiling Lee, Rozieffa Roslan, Shariza Azizan, Mohd Firdaus-Raih, Effirul I. Ramlan

**Affiliations:** 1 Natural Computing Laboratory, Department of Artificial Intelligence, Faculty of Computer Science and Information Technology, University of Malaya, 50603 Kuala Lumpur, Malaysia; 2 School of Biosciences & Biotechnology, Faculty of Science & Technology, and Institute of Systems Biology, Universiti Kebangsaan Malaysia, 43600 Bangi, Malaysia; 3 Bioscience Institute, Universiti Putra Malaysia, 43400 Serdang, Selangor Malaysia; 4 Centre of Research for Computational Sciences and Informatics for Biology, Bioindustry, Environment, Agriculture, and Healthcare (CRYSTAL), University of Malaya, 50603 Kuala Lumpur, Malaysia

**Keywords:** Molecular logic circuit, Molecular programming, RNA computing, Molecular computing, Computational RNA

## Abstract

**Background:**

Biological macromolecules (DNA, RNA and proteins) are capable of processing physical or chemical inputs to generate outputs that parallel conventional Boolean logical operators. However, the design of functional modules that will enable these macromolecules to operate as synthetic molecular computing devices is challenging.

**Results:**

Using three simple heuristics, we designed RNA sensors that can mimic the function of a seven-segment display (SSD). Ten independent and orthogonal sensors representing the numerals 0 to 9 are designed and constructed. Each sensor has its own unique oligonucleotide binding site region that is activated uniquely by a specific input. Each operator was subjected to a stringent *in silico* filtering. Random sensors were selected and functionally validated via ribozyme self cleavage assays that were visualized via electrophoresis.

**Conclusions:**

By utilising simple permutation and randomisation in the sequence design phase, we have developed functional RNA sensors thus demonstrating that even the simplest of computational methods can greatly aid the design phase for constructing functional molecular devices.

**Electronic supplementary material:**

The online version of this article (doi:10.1186/s12859-016-1297-x) contains supplementary material, which is available to authorized users.

## Background

Following the Ebola outbreak in March 2014 [1, 2], Poje et al. [3] demonstrated an alternative diagnostic system comprising of deoxyribozyme-based logic gates that were able to detect the presence of nucleic acid sequences from either a Marburg or Ebola virus. The system generates a read-out (in the form of graphical output) using fluorescent characters (i.e., ‘M’ or ‘E’ denoting the presence of either Marburg and Ebola virus, respectively). This molecular graphical information processing system uses four input oligonucleotides representing the four-bit binary-coded decimal (BCD) values similar to the conventional electronic seven-segment display (SSD) system [4]. When input oligonucleotides bind with their respective deoxyribozymes, this binding triggers the separation of single stranded DNA (ssDNA) from the stem of the deoxyribozyme releasing a product illuminating a fluorescent dye. The separation of the ssDNA is caused by the self-cleavage reaction of the deoxyribozyme [5].

The plausibility of adapting this simple mechanism (as demonstrated by Poje et al. [3]) has been actively investigated [5–7]. Often, the problem of developing these molecular information processors lies in the complexity of sequence to structure relationships [8], where the conformity of the structures is predominantly determined by the combinatorics of vast sets of sequences. Therefore, the programmability of the molecular processors will always be associated with an error margin much larger than the normal error encountered in conventional computers. In most cases, even if the candidate sequences passed an "*in-silico*" filter (i.e., based on simulated profiles generated from computational tools), these candidates are still susceptible to errors during the actual implementation in the laboratory.

The application of RNA molecules as programmable bio-molecules has been extensively investigated [9–11]. RNA is versatile and its ability to function as natural catalysts [12–14] makes them attractive candidates for development as bio-molecular computers [15]. Any system of nucleic acid machines (hybrids of DNA and RNA molecules) can be fabricated and programmed to perform specific tasks. These systems can often be broken down into smaller sub-units that can be constructed individually and then integrated into a functional system [15, 16] similar to a conventional computer system comprised of logic boards, central processing unit (CPU), memory and peripherals.

As we move towards constructing RNA systems that can perform complex logic (i.e., synonymous to the central processing unit), our focus shifts towards generating RNA components that can function as the basic operators of such a system. Logical operations such as identification of delivery site or recognition of specific molecules can be programmed into an RNA component using these operators. For instance, one can imagine Poje et al. [3] logical operations embedded into a DNA structure acting as a carrier with specific functions once an action has been triggered. This hypothetical biomolecular machine can be a product of integration between sub-units, which could become the way forward in fabricating functional biomolecular information processors.

The difficulty of designing and developing practical molecular information processors remains an important issue in the field. Motivated by the prospect presented by Poje et al. [3], we investigated the plausibility of designing RNA sensors using simple permutation and random substitution algorithms. The objective of this work was to provide insights into the practicality and complexity of utilising simple heuristics to aid in the design phase of constructing functional molecular devices.

## Methods

### Molecular Seven Segment display (SSD) design

**Fig. 1:**
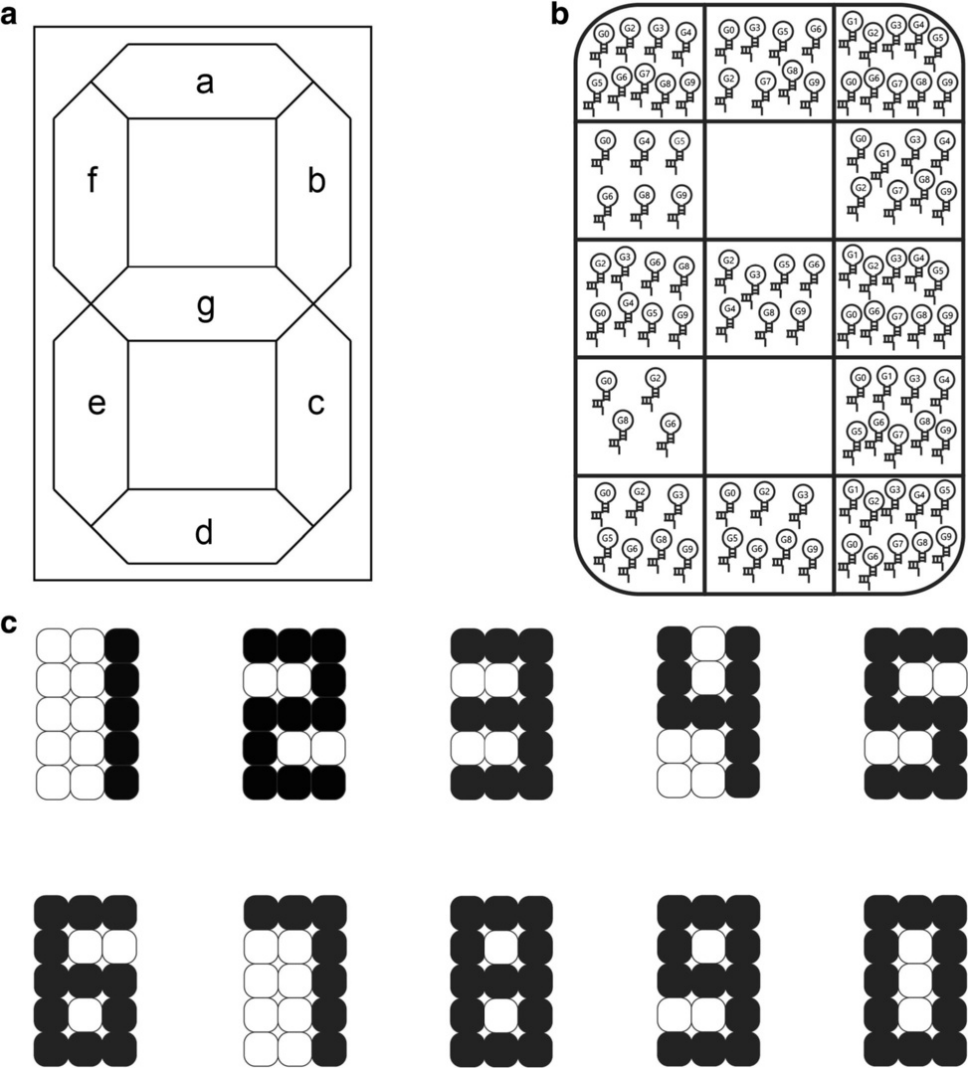
Conceptual representation of molecular SSD. **a** presents the segmentation identification of SSD comprising of 10 individual pins, where each pin can be switched (ON or OFF). Seven of the pins will correlate to the seven LED segments [Refer Additional file for the truth table of the molecular SSD]. **b** illustrates the distribution of logic gates per wells. Each circle represents a gate and gates are distributed accordingly into the wells based on the digits it corresponds to. Wells are labelled as 1 through 15 from left to right and from top to bottom such that the upper left well is labelled as well 1 and the lower right is labelled as well 15. **c** is a conceptual representation of a molecular seven-segment character display. Gates in specific wells will be activated upon binding with its input sequence. For instance, well 1, 2, 3, 6, 9, 12, and well 15 will be activated by the input sequence represent the numerical digit seven (i.e., activation of gate 7)

Seven-segment display (SSD) is a form of graphical display in electronic devices that produces a numeral [17]. SSDs can be found in digital clocks, electronic meters, calculators and other electronic devices that display numeral data. They consist of seven segments with separate sets of combinatorial logic to switch between an ON and OFF state for each segment, and create the required digital output. Each segment of the display is as depicted in Fig. 1 (a). Liquid crystal display (LCD), light emitting diode (LED) or any other light generating mechanism is commonly used as substrates for the SSD.

A standard SSD decoder requires 10 logical states (to display numerals 0 to 9). Accordingly, we can simplify the design of our RNA SSD circuit by separating each state using independent and orthogonal RNA sensors for each input to represent the numerals. If such logic can be supported by a number of basic sub-units, then evidently this logic can be further simplified with a more complicated unit [3]. We implemented 10 RNA sensors to mimic the complete logic of the conventional SSD as illustrated in Fig. 1(b). Conceptually, 15 wells comprising of a collection of 10 RNA sensors that represent a numerical value of 0 through 9 were used in the system. Figure 1(c) shows the activation of the sensors based on Fig. 1(b) to display the numerals 0 through 9.

Penchovsky and Breaker [18] have created a modular form of four universal logical operators using allosterically controlled hammerhead ribozymes (i.e., the AND, OR, YES and NOT Boolean logic gates). These engineered ribozymes are able to demonstrate ligand specificity and were validated successfully in the laboratory. Each allosterically controlled ribozyme logical operator has an interchangeable architecture that allows computational alteration to be made to the oligonucleotide binding site (OBS) region without altering its unique allosteric function and conformation. In this work, the YES-1 gate [18] was selected as a reference model for our RNA sensor design.

Conventionally, to construct a molecular circuit (i.e., molecular array of YES and AND gates [15, 19]), multiple hammerhead ribozymes are placed in wells according to their corresponding logic. Each operator has a specific oligonucleotide input. Upon the presence of their respective input, the self-assembly process between input oligonucleotide and ribozymes occur, facilitating the conformational change that will permit the self-cleavage reactions to happen. The conformation change from inactive to active state is a representation of an ON value. For instance, to display the number one (1), only input for sensor representing numeral 1 will be inputted into each well. Wells with sensor representing numeral 1 will undergo catalytic reaction thus effecting a change of state (from OFF to ON). By combining the wells (corresponding to each segment), the RNA SSD will display the correct numerals. Thus any mismatch binding (identified as error) in the wells may lead to the failure of the sensor.

### Candidate generation using simple computational pipeline

The pipeline to generate these candidate sequences comprise of two steps: (I) generation of random sequences using three randomisation strategies and; (II) selection of candidates using an *in silico* filtering cascade. The computational pipeline is straightforward to implement; step I is executed repeatedly, followed by step II once step I has been completed (i.e., generate candidate repeatedly, and discard the candidate if it does not pass the filter cascade). Meta-heuristics are not required in the pipeline as the generation of the candidates can be viewed solely as a repetitive cycle (i.e., non-converging).

**Fig. 2:**
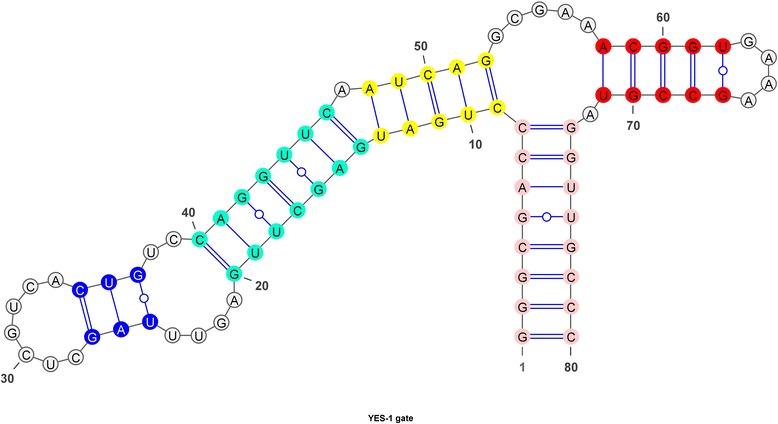
The dependency diagram for YES gate. The colours represent the inter-binding of each base position. These coloured bases are interdependent where changing one base should change its complementary base in order to retain the secondary structure whereas the white bases indicate that the base is not complementary or binds to any base position

The three strategies implemented in step I are based on the dependency diagram [20] of each base position for the two meta-stable states (inactive and active conformations). The dependency diagram provides a relational outlook of each base and allows substitution of bases to be made according to the severity of the base pairing interferences in the two meta-states. Each strategy represents the differences in severity level of substituting specific regions of the sequences (from minimal to loose) based on the dependency of each base (to be paired or unpaired; Fig. 2) in the two meta-states. Note that the mutations that are suggested by the algorithm must comply with the conformational integrity indicated in the dependency diagram. Therefore, the selection of the base positions is restricted to the independent ("free") position where the bases are predicted to cause minimal changes to the structural integrity. Only these positions were selected for the first strategy (9 base positions) and the second strategy (7 base positions).

### Generate random sequences (Step I)

#### First strategy

**Fig. 3:**
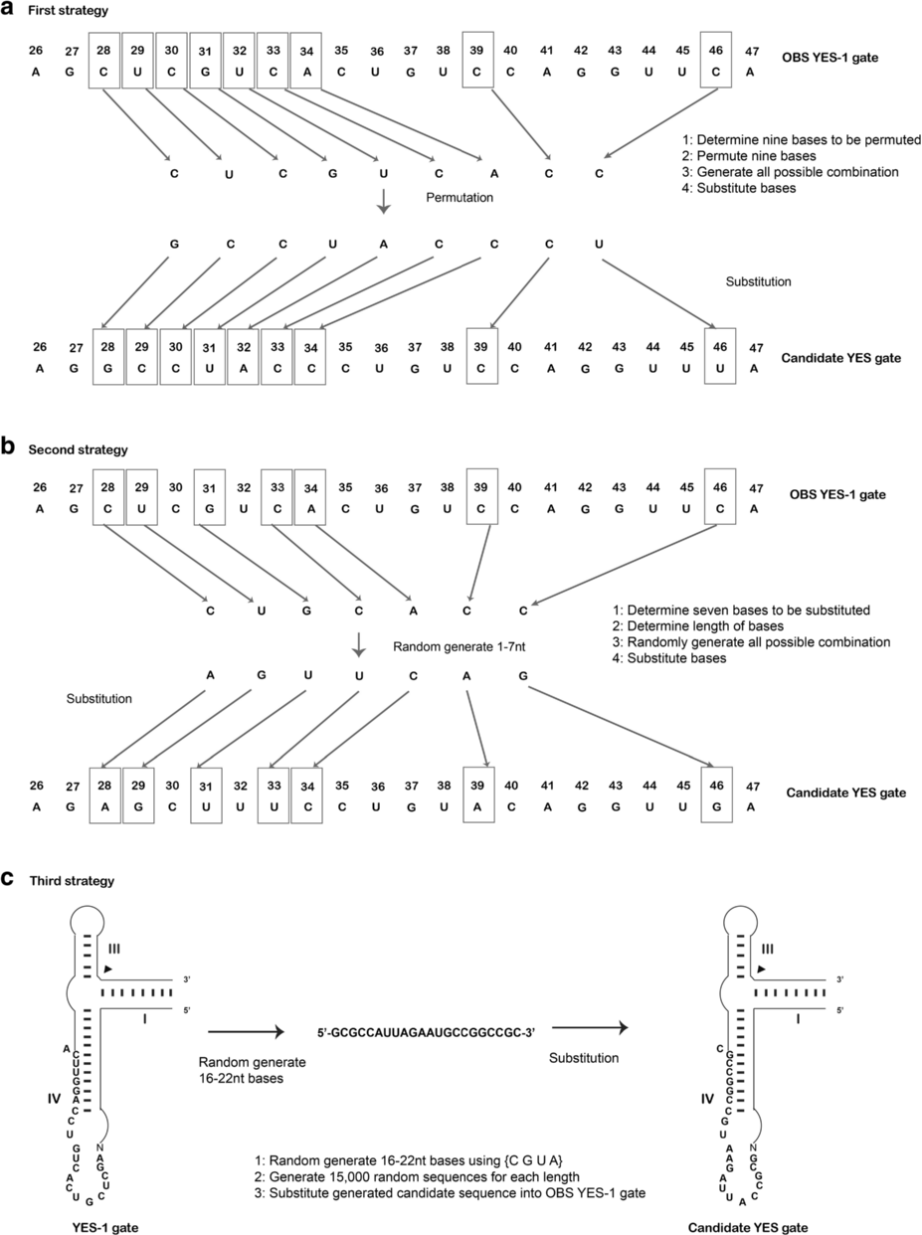
Schematic representation of the three algorithms for logic gate design. **a** First strategy: the steps to permute nine bases within the OBS YES-1 gate. **b** Second strategy: the steps to perform random substitutions of seven bases within the OBS YES-1 gate and **c** Third strategy: substitutions of the complete OBS YES-1 sequence

Mutations are permitted at nine base positions. Nucleotide C28, U29, C30, G31, U32, C33, A34, C39 and C46 were permuted. All possible combinations were generated using the procedure below (refer Fig. 3a). Because there are only four nucleobases namely Adenine (A), Uracil (U), Guanine (G) and Cytosine (C) in the permutation list, there will be repetitive strings for the candidate sequences, therefore only unique strings are permitted (i.e., removal of repetitions). This strategy was considered to be the most stringent as it only alters non-binding bases and the substitution of bases will only be selected from a list of distinct permuted strings.

1. Determine the nine bases in OBS that have to be permuted;
2. Generate the permutation list on the nine positions;
3. Generate all possible combinations of bases;
4. Substitute the bases within the OBS accordingly.

#### Second strategy

Random generation of strings with 1–7 nucleotides (*nt*) in length to be substituted into the following seven positions (C28, U29, G31, C33, A34, C39 and C46) (refer Fig. 3b). These random strings were then substituted to the original OBS of the YES-1 gate at positions 28, 29, 31, 33, 34 and 46 thus elongating the OBS region to be in the range of 16 to 22 *nt*.

1. Determine length/number of bases to be substituted (the length should be between 1 and 7 *nt*);
2. Random generation of characters from {C, G, U, and A} according to the number of bases defined;
3. Generate all possible combinations of bases;
4. Substitute the bases within the OBS accordingly.

#### Strategy 3

Randomly generated sequences were used to substitute the OBS region of the YES-1 gate (refer Fig. 3c). The length of the sequences was in the range of 16 to 22 *nt*. As the number of possible combinations of nucleotides is 4^*n*^ (where *n* = 16 to 22), the generation of random sequences was restricted to only 15,000 for each length. The generated sequences then replaced the original OBS region. This approach liberated the constraints, thus enlarging the search space to find more plausible solutions to construct a richer pool of candidate sequences.

1. Randomly generate strings with 16–22 *nt* bases by using {C, G, U and A} to substitute the OBS region of the YES-1 gate;
2. Generate 15,000 random sequences for each length;
3. Combine the upper core sequences of the YES-1 gate (GGGCGACCCUGAUGAGCUUGAGUUU) to the newly generated OBS sequences and lower core sequences (AUCAGGCGAAACGGUGAAAGCCGUAGGUUGCCC) to construct sequences that are approximately 80 *nt* in length.

### Selection of candidates (Step II)

In step II, computational analysis was conducted to select plausible candidates according to the filter cascade recommended for wet-lab validation [18]. The secondary structures and minimum free energy (MFE) for each sequence were calculated using *RNAfold* [21] with the Turner 1999 energy model [22] and Turner 2004 energy model [23]. Candidate sequences that do not fold into an OFF meta-state conformation using direct secondary structure (dot-bracket) comparison were eliminated from the pool. A similar procedure was applied to check the conformation of the candidate sequences for the ON meta-state conformation. The dominant structures of the candidate sequences were determined using dot matrix plots derived from the partition function calculations [24]. Similarly, nonconforming candidates were eliminated. Next, each candidate was subjected to a filter cascade following this criteria:

- Sequence must not have more than four consecutive identical bases;
- A total of 30 to 70 % of nucleotides in the oligonucleotide binding site (OBS) must participate in base pairing interactions in the absence of the DNA facilitator (input strand);
- The free energy gap between the OFF meta-state and the ON meta-state must be in the range of -6 to -10 kcal/mol;
- The dominant structures of the ON and OFF meta-states must be preserved in the range of 20 to 40 °C;
- The ensemble diversity values for both the ON and OFF meta-states must be less than nine base pairs.

The ensemble diversity indicates the average base pair distance between RNA suboptimal structures as predicted by *RNAfold* [21]. Ideally, the ensemble diversity values should be minimized. The threshold was set to less than nine base pairs as suggested by Penchovsky and Breaker [18]. Additionally, we also considered the percentage of GC pairing in the OBS region. The number of G and C bases in the OBS region was counted, and only candidates with more than 50 % of G and C base pairs were selected. Base pair formations must be with other bases outside of the region.

We define *V* as the penalty value of each candidate sequence. The value of each candidate can be calculated by, *V* = ∑_*i* = 1_^6^(*C*_*i*_)/6 where C_*i*_ is the score of each criterion as described in Table 1. The value C_*i*_ is equal to 1 if a given sequence satisfies the criterion or 0 otherwise. The value of *V* for each sequence is equal to the sum of the C_*i*_ divided by six (i.e., average score of the six filters). The threshold value for *V* was set to 0.9 for stringent quality control. The program *RNAsuboptimal* [25] was utilized to verify the presence of both OFF and ON meta-states using the two energy parameters (Turner 1999 energy model and Turner 2004 energy model) for each sequence. The free energy folding parameter between suboptimal conformations was set to 1 kcal/mol.

**Table 1:** Description of the filter cascade criteria (*C*_*i*_). The scoring system to filter the candidates is an average score of six criteria as listed in the table. Scores are given only if the candidates fulfil the criterion. Otherwise, a penalty score of 0 will be assigned

Descriptions	Score
Having not more than 4 identical consecutive nucleotide	C_1_
Remain inactive state without input oligonucleotide	C_2_
Percentage of OBS participate in base pairing	C_3_
Ensemble diversity for both ON and OFF state	C_4_
Free energies gap between ON and OFF state	C_5_
Percentage of GC pairing in OBS	C_6_

### Protocol for laboratory validation

#### Oligonucleotides

Synthetic RNAs (Gate) and DNAs (Input) were designed and modified according to Penchovsky and Breaker [18]. These synthetic RNAs were converted into DNA templates with the insertion of the T7 promoter sequence at the 5’ end. The high-performance liquid chromatography (HPLC) and desalted purified DNA were purchased from Aitbiotech (Singapore).

#### Transcription

RNA synthesis was carried out by in vitro transcription using the MEGAshortscriptTM Kit (Ambion, USA) in a 20 μl final volume according to the manufacturer’s instruction. The reaction mixtures consisted of 2 μl T7 10× reaction buffer, 2 μl T7 ATP solution (75 mM), 2 μl T7 CTP solution (75 mM), 2 μl T7 GTP solution (75 mM), 2 μl T7 UTP solution (75 mM), mixture of 1 μg DNA templates (DNA Gate and T7 promoter), 2 μl T7 enzyme mix, and nuclease free water. The reaction mixtures were incubated for 4 h at 37 °C in a Mastercycler® Gradient (Eppendorf, Germany) thermocycler to initiate the transcription reaction. The DNA templates were then removed from the mix by adding 2.5 μl DNase I (Qiagen, Germany), 10 μl RDD5 and RNase free water to the final volume of 100 μl for each sample and incubated at 25 °C for 30 min.

#### RNA purification

Termination of the reaction and RNA recovery were carried out by alcohol precipitation. 115 μl of nuclease free water (Ambion, USA) and 15 μl of 3 M sodium acetate were added into the reaction mixtures and mixed thoroughly. Next, two volumes of ethanol were added (±300 μl), mixed well then chilled at -20 °C for at least 15 min. The RNA was pelleted for recovery by centrifugation at 4 °C for 15 min at 12,000 g using a Microfuge 22R centrifuge (Beckman Coulter). The supernatants were removed and the RNA pellets were suspended in OmniPur Water (Calbiochem, USA). The purified RNA was analysed via electrophoresis in 20 % denaturing PAGE (0.5 ml of 10× TBE, 2.4 g 8 M urea, 2.5 ml of 40 % acrylamide, nuclease free water, 30 μl of 10 % APS, 3 μl TEMED) in 1× TBE buffer at 50 V and a low molecular weight DNA ladder (New England Biolabs® Inc, Massachusetts) was used as a migration distance reference. The RNA concentration was quantified using a NanoDrop2000 (Thermo Scientific, USA) spectrophotometer.

#### Ribozyme assay

The ribozyme activity assays were performed by mixing 0.1 M Tris-HCl, 1 μM RNA transcript, 6 μM DNA input, ddH_2_O and 0.02 μM MgCl2 in 10 μl of total reaction volume. The reaction mixtures were incubated at 25 °C in a Mastercycler Gradient (Eppendorf, German) thermocycler for 2 h. The self-cleavage reactions were stopped using one volume of stop buffer containing loading dye (bromophenol blue) and 0.5 M Ethylenediaminetetraacetic acid (EDTA). The samples were denatured at 95 °C for 2 min and the results of the ribozyme reaction were analysed by electrophoresis in 20 % denaturing polyacrylamide gel.

#### Visualization

The polyacrylamide gel was silver stained in order to visualize the separation of the RNA molecules. Immediately after the electrophoresis, the gel was placed into a container and fixed with 40 % methanol for at least 30 min, followed by the oxidizer for five minutes. A large volume of sterile water was added to flush the orange stain for a maximum of 15 min. Next, the gel was immersed in silver reagent for 20 min followed by a quick water rinse to wash off the left-over silver reagent. The gel was then immersed developer solution until the desired intensity of the band was observed and the developer reaction was stopped by addition of a 5 % acetic acid solution. The finished gel was viewed and photographed using an Alpha Imager.

## Results and discussion

### Analysis of *in silico* results

**Fig. 4:**
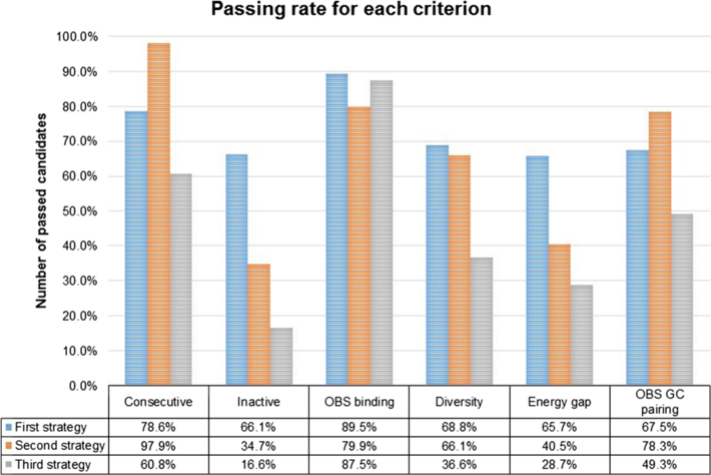
The passing rate of candidates for each criterion. Six criteria (not more than three consecutive nucleotides, present in the inactive state, 30*–*70 % OBS binding, diversity value not more than 9, having energy gap within -6 to -10 kcal/mol and have at least 58 % OBS GC pairing) were used to select the candidate sequences

The first strategy produced a total of 362,880 sequences. After the removal of identical sequences, only 1511 unique sequences remained. The step II filtering yielded only 500 candidates that fulfilled all the criteria. The second strategy produced 26,633,664 candidates of which only 49,849 unique sequences remained after identical sequences were eliminated. From the 49,849 candidates, only 5262 candidates passed the step II filter. In the third strategy, a total of 105,000 sequences were generated (after the consideration of search space, 15,000 candidates for each length). After step II screening, only 2559 sequences remained. Further analysis was conducted to find overlapping candidates from the three strategies, however, no identical candidates were found from the pool of 8321 candidates that progressed past the step II screen. The distribution of candidates for each criterion is presented in Fig. 4.

As depicted in Fig. 4, although the third strategy generated the largest number of candidates, the overall passing rate of the third strategy is only 2.4 %. In contrast, the first strategy (i.e., strict mutation strategy) produced the highest overall passing rate of 33.1 %, despite having the least number of candidates. The second strategy has 5262 candidates with the passing rate of 10.5 %. Of all the criteria, the passing rate is largely dependent on the ability of candidates to remain in its inactive secondary structure during initial folding. For instance, although we successfully generated 105,000 candidates using the third strategy, only 17,465 (16.6 %) candidates remained in its inactive secondary structure after initial folding, causing a significant reduction of the potential candidates after criterion 1. Candidates from the first strategy performed better with 66.1 %, followed by candidates from the second strategy with 34.7 %. This gives a strong indication that the randomisation of the length and base combinations in the OBS region plays an essential role in the formation of a stable OFF state conformation. Deviation from the original configuration had a significant impact on the sensor design.

As a result, it is simple to generate conforming sequences when the number of candidates is limited as indicated by the 500 candidates from the first strategy. However, the limited number of candidates increased the homogeneity of the sequences which led to the increase of the mispairing probability between each input and sensor unit. The liberation of the dependency constraints fixed this issue as exemplified by the the third strategy. As a consequence, nonconformity of the candidates increase. Only 2558 successful candidates were obtained from the largest pool of plausible candidates generated by the third strategy after step II. Followed by 5262 candidates from the second strategy. Further analysis revealed that the candidates from these two strategies possessed a better mixture of bases combination. This is an important factor in increasing the potential of avoiding mispairing, especially when the number of sensors required for the system increases. Identifying the trade-offs between conformity and sequence diversity is key in generating plausible candidates for any type of sensor.

Analysis of the successful candidates also revealed the importance of determining key dependent base positions (conserved bases) previously undiscovered during dependency analysis. Although we permute and randomly change the nucleotide in the OBS YES-1 gate (in first strategy and second strategy), the base position N46 can only be assigned with either base C or U. The presence of either base G or A at position N46 will immediately activate the sensor. Therefore, detail anaysis of the information generated from the dependency graph is imporant to better guide the randomisation strategy.

### Analysis of in vitro validation

Wet laboratory validation was conducted on 10 random samples selected from each strategy. For the first strategy, the sizes of all sensors were fixed at 80 *nt* as permutation was limited to only the nine existing base positions. However, the sizes varied for the second and third strategies. The selected candidates for the second strategy comprised of three sensors at 80 *nt*, two sensors at 79 *nt*, two sensors at 78 *nt,* two sensors at 87 *nt* and one remaining sensor at 76 *nt*. Meanwhile, for third strategy, we had two sensors at 80 *nt*, one sensor at 79 *nt*, one sensor at 78 *nt,* two sensors at 77 *nt*, one sensor at 76 *nt,* one sensor at 75 *nt* and two sensors at 74 *nt*. The HPLC-purified DNA template lengths were in the range of between 95 to 100 *nt*.

The characteristics of the randomly selected sensors are depicted in Table 2 (for the first strategy), in Table 3 (for the second strategy) and in Table 4 (for the third strategy). Information regarding the sequences of the selected sensors is available in the supplementary materials (See Additional file 1: S2: Sequences for candidates from the second strategy and S3: Sequences for candidates from the third strategy). Input sequences for the candidates from the third strategy were excluded due to the non-conforming structure formation of each sensor during the transcription process.

**Table 2:** The characteristics of the randomly selected YES gates from the first strategy. Gate numbers are from 0 to 9 (10 random gates). The first column is the minimum free energy (MFE) value of the candidate as predicted by the program *RNAfold*. The similarity percentage between the benchmark YES-1 gate (18) with each candidate is represented in the next column. This similarity analysis is localized to only the OBS region (i.e., Bases in the OBS region are aligned and counted). The percentage of OBS binding is basically the number of paired bases in the OBS region (based on the prediction of the meta-stable states that form the inactive conformation). The dissimilarity between the benchmark structure (YES-1 in (18)) and the candidates is represented as an average base pair distance (*bpdistance*) value calculated using the program *RNAdistance*. The base pair distance represents the average number of mutational steps required for the candidates to form identical conformation with a given benchmark structure. The ensemble diversity represents the base pair distances among sub-optimal structures predicted from the candidate sequences. A lower ensemble diversity value indicates a more stable conformation of sequence

Gate	MFE kcal/mol	Percentage of similarity	Percentage of binding (OBS)	Base-pair distance	Ensemble diversity
0	−36.80	93	59.09	6	7.5
1	−38.30	94	59.09	7	5.18
2	−35.40	94	59.09	8	6.04
3	−36.10	95	59.09	8	8.13
4	−35.60	95	50.00	11	3.87
5	−35.70	96	68.18	8	6.63
6	−37.10	91	59.09	6	4.74
7	−36.30	93	54.55	0	3.51
8	−36.21	94	50.00	9	7.23
9	−37.20	93	63.64	1	2.77

**Table 3:** Characteristics of the randomly selected YES gates from the second strategy. The characteristics are similar to Table 1 with the exception of the base pair distance column. Although the base positions that are permissible for mutation are localized to ensure non-interferences of the confirmation, the base pair distance information is no longer necessary as the size of the localized region is now random and no longer complies with the size suggested for the benchmark YES-1 gate (18)

Gate	MFE kcal/mol	Percentage of similarity	Percentage of binding (OBS)	Ensemble diversity
0	−34.10	94 %	68.75	6.99
1	−31.20	93 %	68.75	6.62
2	−33.40	95 %	68.75	5.98
3	−31.40	93 %	50.00	4.37
4	−33.60	94 %	68.75	6.28
5	−33.10	93 %	68.75	6.08
6	−32.00	91 %	43.75	8.00
7	−31.00	91 %	50.00	4.25
8	−34.30	93 %	68.75	4.40
9	−33.30	91 %	50.00	4.31

**Table 4:** Characteristics of the randomly selected YES gates from the third strategy. The characteristics are similar to Table 1 with the exception of the base pair distance column. The column is excluded because the mutations allowed in for candidates in the third strategy are no longer bound by the constraints previously imposed in the first and second strategies. The base positions permissible for mutation are no longer restricted

Gate	MFE kcal/mol	Percentage of similarity	Percentage of binding (OBS)	Ensemble diversity
0	−34.10	9 %	50 %	4.64
1	−34.90	27 %	50 %	6.4
2	−34.60	23 %	41 %	4.36
3	−34.70	18 %	50 %	5.8
4	−34.80	23 %	47 %	4.69
5	−34.40	36 %	47 %	5.6
6	−33.70	27 %	35 %	5.18
7	−34.60	32 %	38 %	7.34
8	−34.40	27 %	55 %	7.35
9	−35.40	77 %	59 %	6.04

**Fig. 5:**
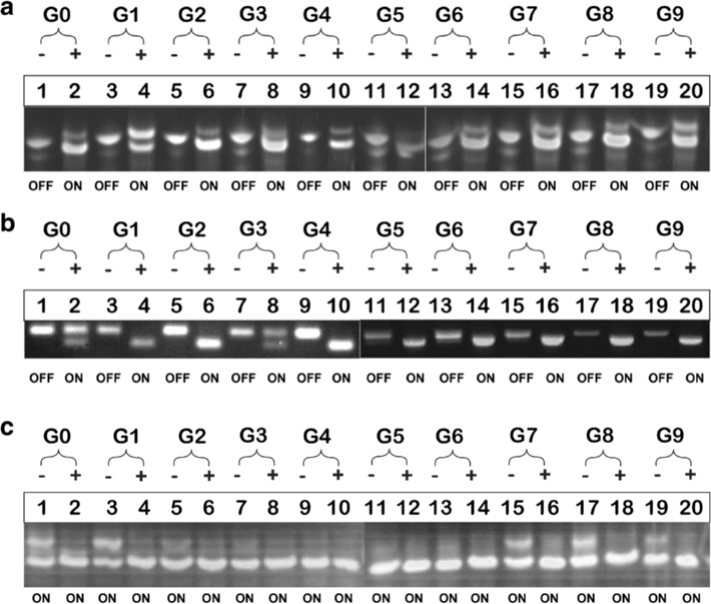
Profile of ribozyme assays visualized in 10 % denaturing PAGE at 59 V. Gate activity without the complementary input oligonucleotides (-) and with the presence of complementary input oligonucleotides (+) is showed. The smears beneath each band show immature transcripts of DNA. This does not influence the self-cleavage reaction. Figure 5 shows the self-cleavage activity for candidates generated by (**a**) first strategy (**b**) second strategy and (**c**) third strategy

Figures 5(a) and (b) depict the self-cleavage activity of candidate sensors from the first and second strategies respectively. Each sensor was activated by its complementary input oligonucleotide and remained inactive in the absence of the input. This is in contrast to Fig. 5(c), where activation of the sensors occurred without the presence of the input. Sensors from the third strategy were unable to fold into an inactive conformation because the dependent bases remained free (i.e., failed to form base pairs with the bases in the OBS region). The random base substitution affecting all bases in the OBS region failed to function as predicted in step II. During wet-lab validation, the binding between bases in the OBS region and the conserved bases were not thermodynamically favourable.

**Fig. 6:**
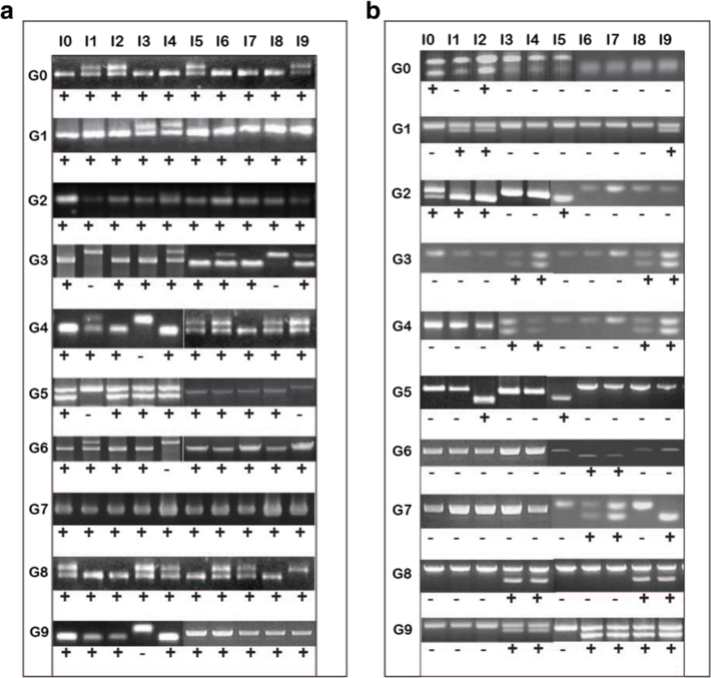
Mismatch profile for candidate gates**.** This figure presents the activity of the gates for the first (**a**) and second strategy (**b**) with the insertion of ten input DNA oligonucleotides that were visualized in 10 % denaturing PAGE at 59 V Input 0 through input 9 were inserted into each well respectively. Each well depicting double bands indicates the occurrence of cleavage reaction. The highlighted cells represent the gate with its complementary input sequences. Character ‘X’ indicates mismatches and cells without an “X” indicate no mismatch observed

In order to perform the system integration of the sensors, a mismatch profile for all candidates were generated. A mismatch pairing between the input and OBS region can cause sensor inactivation and may lead to the activation of incorrect sensors (i.e., cross-reactivity between sensors). Using the mismatch profile, we were able to validate the specificity of each sensor. We added all effector DNA oligonucleotides (input) to each sensor and as depicted in Fig. 6a, mispairings were present across all sensors (candidates from the first strategy). For several inputs not only did they bind to their respective sensors but also to other sensors. As indicated in the figure, the specificity of input 0 (I0), input 2 (I2), input 5 (I5), input 6 (I6) and input 7 (I7) are not preferable due to the higher cross-talk reactions with other sensors. Accordingly, the self-cleavage activity can be detected in all sensors.

Generally, mismatches occur because of the homogeneity of the candidate sequences. For the first strategy, the sequence similarity percentage is high across the candidate sequences. This is expected as the generation of the candidates was achieved by only substituting (permute) nine base positions within the OBS region alone. The remaining 71 nucleotides were unchanged, which is approximately only 11 % of the region for each candidate. Multiple sequence alignments of the candidate sequences revealed that two bases (A and G) occupied this region in the majority of the candidates. By restricting the nucleotide bases to these nine bases, we had significantly reduced the specificity between the candidate sequences resulting in them having a high percentage of base combination similarity.

We observed that the rate of mismatches for the second strategy has been reduced to less than 50 % as compared to the first strategy (Fig. 6b). The number of mismatches dropped from 84 mismatches to only 36 mismatches. Among these ten input sequences, input 5 (I5) and input 6 (I6) were the most specific. The I5 will only activate sensor 2 and its respective sensor, while I6 will only activate sensor 7 and its respective sensor. The input 9 (I9) had the lowest specificity in being able to activate six sensors. The remaining inputs were able to activate three to four other sensors including their respective sensors. When compared to the first strategy, the substitution region for each candidate is larger depending on the size of the elongated region (which varies from 16 to 22 *nt*). This allows for better diversity of sequence combinations to be achieved. Multiple sequence alignments revealed that an almost equal bases distribution occurred across all candidates from strategy two in the region of interest. This also indicated the availability of better candidates to be selected for validation from the pool generated by the second strategy.

From the mismatch profile experiment, it is evident that the specificity factor of the candidate sequences has to be considered during the design phase. However, embedding the sophistication of the cross-reactivity analysis (or inter-dependant base pairing) as a function in the substitution strategy would greatly increase the computational complexity of the heuristic. The complexity would become quadratic, as the heuristic would perform pairwise checks for cross-reactivity between all pairs of gates. On the one hand, as it is, a simple and crude strategy would not be able to produce sensors with adequate specificity that can ensure reliable activation. On the other, the results proved that enriching the candidate sequences using the simplified strategies is attainable.

In order to improve the generation of candidates, we have to thoroughly investigate the structural and sequence characteristic of the reference model. In this study, the design of the sensor is solely based on the YES-1 gate [18] to ensure the functionality of the sensor in the laboratory. Base substitutions were restricted to preserve the structural integrity and as a result, the strategy produced homogenous candidates as revealed in our findings. This reference model has to be extended to include structural variations by either elongating non-participating regions or sequence mutations (i.e., substitution of base pairs in the non-active regions) to improve the diversity of the candidates. Enriching the reference model allows for heterogeneous candidates to be generated while to a certain extent preserving the conformational integrity (i.e., good mixture of conserved and permissible base positions). In addition, a cross-reactivity filter to check on the base composition of the regions of interest should be added to further improve on the binding specificity.

## Conclusions

The fabrication of functional molecular devices is synonymous with sophisticated heuristics for designing candidate sequences, complex inter-molecular reaction analysis, and excessive laboratory experiments. There should be a more accessible approach in constructing these functional devices because of their beneficial potential applications. Due to the advancement of computers, we are no longer restricted to a localized search landscape when generating sequences. The analysis presented in this study allows for a simple algorithm to be exploited, with the potential to create a library of molecular components without many restrictions. This reduces the burden of generating compatible candidates and allows for better structural designs as well as more effective functional mechanisms to be implemented during the design phase.

Smarter heuristics and molecular design schematics will enable the extensions of this simple approach to produce better operators thus reducing the complication of wet-laboratory experimentation normally required in constructing these devices.

## Additional file

Additional file 1:
**S1.** The truth table of seven-segment character display. Alphabets (a-g) represent the seven segment of SSD. Each of the digits will be displayed based on the combination of current flows in the seven segments. **S2.** Input oligonucleotides (first strategy). Input sequences to activate the respective sensors generated from the first strategy. **S3.** Input oligonucleotides (second strategy). Input sequences to activate the respective sensors generated from the second strategy. (DOC 44 kb)

## Abbreviations

MFE: Minimum free energy
*nt*: Nucleotide
OBS: Oligonucleotide binding site
SSD: Seven segment display
ssDNA: Single stranded DNA

## References

[1] Bausch DG, Schwarz L (2014). Outbreak of Ebola virus disease in Guinea: where ecology meets economy. PLoS Negl Trop Dis.

[2] Gire SK, Goba A, Andersen KG, Sealfon RSG, Park DJ, Kanneh L, Jalloh S, Momoh M, Fullah M, Dudas G, Wohl S, Moses LM, Yozwiak NL, Winnicki S, Matranga CB, Malboeuf CM, Qu J, Gladden AD, Schaffner SF, Yang X, Jiang P-P, Nekoui M, Colubri A, Coomber MR, Fonnie M, Moigboi A, Gbakie M, Kamara FK, Tucker V, Konuwa E, Saffa S, Sellu J, Jalloh AA, Kovoma A, Koninga J, Mustapha I, Kargbo K, Foday M, Yillah M, Kanneh F, Robert W, Massally JLB, Chapman SB, Bochicchio J, Murphy C, Nusbaum C, Young S, Birren BW, Grant DS, Scheiffelin JS, Lander ES, Happi C, Gevao SM, Gnirke A, Rambaut A, Garry RF, Khan SH, Sabeti PC (2014). Genomic surveillance elucidates Ebola virus origin and transmission during the 2014 outbreak. Science.

[3] Poje JE, Kastratovic T, Macdonald AR, Guillermo AC, Troetti SE, Jabado OJ, Fanning ML, Stefanovic D, Macdonald J (2014). Visual displays that directly interface and provide read-outs of molecular states via molecular graphics processing units. Angew Chem Int Ed.

[4] Patrick DR, Frado SW (2008). ebrary Academic Complete. Electricity and Electronics Fundamentals. Second edition.

[5] Stojanovic MN, Mitchell TE, Stefanovic D (2002). Deoxyribozyme-based logic gates. J Am Chem Soc.

[6] Qian L, Winfree E (2011). A simple DNA gate motif for synthesizing large-scale circuits. J R Soc Interface.

[7] Soukup GA, Breaker RR (1999). Nucleic acid molecular switches. Trends Biotechnol.

[8] Ramlan EI, Zauner KP. In-silico design of computational nucleic acids for molecular information processing. J Cheminform. 2013;5(1):22. doi:10.1186/1758-2946-5-22. 10.1186/1758-2946-5-22 PMC3664215 23647621

[9] Isaacs FJ, Dwyer DJ, Collins JJ (2006). RNA synthetic biology. Nat Biotechnol.

[10] Win MN, Smolke CD (2008). Higher-order cellular information processing with synthetic RNA devices. Science.

[11] Qiu M, Khisamutdinov E, Zhao Z, Pan C, Choi J-W, Leontis NB, Guo P (2013). RNA nanotechnology for computer design and in vivo computation. Phil Trans R Soc A.

[12] Allison LA (2007). Fundamental molecular biology.

[13] Kruger K, Grabowski PJ, Zaug AJ, Sands J, Gottschling DE, Cech TR (1982). Self-splicing RNA: autoexcision and autocyclization of the ribosomal RNA intervening sequence of tetrahymena. Cell.

[14] Prody GA, Bakos JT, Buzayan JM, Schneider IR, Bruening G (1986). Autolytic processing of dimeric plant virus satellite RNA. Science.

[15] Stojanovic MN, Stefanovic D (2003). A deoxyribozyme-based molecular automaton. Nat Biotechnol.

[16] Ong HS, Rahim MS, Firdaus-Raih M, Ramlan EI (2015). DNA tetrominoes: the construction of DNA nanostructures using self-organised heterogeneous deoxyribonucleic acids shapes. PLoS One.

[17] Moyer NE, Walker DE (1975). Segment decoder for numeric display. Google Patents.

[18] Penchovsky R, Breaker RR (2005). Computational design and experimental validation of oligonucleotide-sensing allosteric ribozymes. Nat Biotechnol.

[19] Macdonald J, Li Y, Sutovic M, Lederman H, Pendri K, Lu W, Andrews BL, Stefanovic D, Stojanovic MN (2006). Medium scale integration of molecular logic gates in an automaton. Nano Lett.

[20] Ramlan EI, Zauner K-P (2011). Design of interacting multi-stable nucleic acids for molecular information processing. BioSyst.

[21] Lorenz R, Bernhart SH, Höner zu Siederdissen C, Tafer H, Flamm C, Stadler PF, Hofacker IL (2011). Vienna RNA package 2.0.. Algorithms Mol Biol.

[22] Mathews DH, Sabina J, Zuker M, Turner DH (1999). Expanded sequence dependence of thermodynamic parameters improves prediction of RNA secondary structure. J Mol Biol.

[23] Mathews DH, Disney MD, Childs JL, Schroeder SJ, Zuker M, Turner DH (2004). Incorporating chemical modification constraints into a dynamic programming algorithm for prediction of RNA secondary structure. Proc Natl Acad Sci U S A.

[24] McCaskill JS (1990). The equilibrium partition function and base pair binding probabilities for RNA secondary structure. Biopolymers.

[25] Wuchty S, Fontana W, Hofacker IL, Schuster P (1999). Complete suboptimal folding of RNA and the stability of secondary structures. Biopolymers.

