# Supplementary material for: An analysis of simple computational strategies to facilitate the design of functional molecular information processors
Source: BMC Bioinformatics. 2016 Oct 28;17:438. doi: 10.1186/s12859-016-1297-x (PMC5086070; doi:10.1186/s12859-016-1297-x)
Supplement: Additional file 1: — S1. The truth table of seven-segment character display. Alphabets (a-g) represent the seven segment of SSD. Each of the digits will be displayed based on the combination of current flows in the seven segments. S2. Input oligonucleotides (first strategy). Input sequences to activate the respective sensors generated from the first strategy. S3. Input oligonucleotides (second strategy). Input sequences to activate the respective sensors generated from the second strategy. (DOC 44 kb) [file 12859_2016_1297_MOESM1_ESM.doc]

**Additional file**

**The truth table of seven-segment character display**

Alphabets (a-g) represent the seven segment of SSD. Each of the digits will be displayed based on the combinations of current flows in the seven segments.

| **Digit** | **a** | **b** | **c** | **d** | **e** | **f** | **g** |
| --- | --- | --- | --- | --- | --- | --- | --- |
| 1 | 0 | 1 | 1 | 0 | 0 | 0 | 0 |
| 2 | 1 | 1 | 0 | 1 | 1 | 0 | 1 |
| 3 | 1 | 1 | 1 | 1 | 0 | 0 | 1 |
| 4 | 0 | 1 | 1 | 0 | 0 | 1 | 1 |
| 5 | 1 | 0 | 1 | 1 | 0 | 1 | 1 |
| 6 | 1 | 0 | 1 | 1 | 1 | 1 | 1 |
| 7 | 1 | 1 | 1 | 0 | 0 | 0 | 0 |
| 8 | 1 | 1 | 1 | 1 | 1 | 1 | 1 |
| 9 | 1 | 1 | 1 | 1 | 0 | 1 | 1 |
| 0 | 1 | 1 | 1 | 1 | 1 | 1 | 0 |

Input oligonucleotides for gates from the first strategy.

| Gate | Input oligonucleotide |
| --- | --- |
| 0 | TGAACCTGCACAGAAGGGTGCT |
| 1 | TGAACCTGAACAGGGCAGTGCT |
| 2 | TGAACCTGGACAGACTAGGGCT |
| 3 | TGAACCTGGACAGTACAGGGCT |
| 4 | TGAACCTGGACAGGGCATAGCT |
| 5 | TGAACCTGGACAGGGATCAGCT |
| 6 | TGAACCTGTACAGAGGGAGCCT |
| 7 | TGAACCTGGACAGACGGGTACT |
| 8 | TGAACCTGGACAGGGAGCTACT |
| 9 | TAAACCTGTACAGCGGAGGGCT |

Input oligonucleotides for gates from the second strategy.

| Gate | Input oligonucleotide |
| --- | --- |
| 0 | TGAACCTGAACAGTCATGGTCT |
| 1 | TAAACCTGTACAGCTAAGTTCT |
| 2 | TGAACCTGTACAGTTACGGACT |
| 3 | TGAACCTGACAGGAGGCGCT |
| 4 | TGAACCTGACAGTTAGTTCT |
| 5 | TGAACCTGTACAGCCAGCT |
| 6 | TAAACCTGACAGCTAGTCT |
| 7 | TAAACCTGACAGAATGCT |
| 8 | TGAACCTGACAGTCAGCT |
| 9 | TGAACCTGACAGAGACT |
